# Spontaneous rupture of renal angiomyolipoma and its management: A case report

**DOI:** 10.1016/j.amsu.2022.104037

**Published:** 2022-06-18

**Authors:** Narayan Thapa, Suman Maharjan, Anil Hona, Jayaram Pandey, Sabin Karki

**Affiliations:** a Department of Surgery, Shree Birendra Hospital, Chhauni, Kathmandu, 44600, Nepal; b College of Medicine, Nepalese Army Institute of Health Sciences (NAIHS), Sanobharyang, Kathmandu, 44600, Nepal

**Keywords:** a) Case report, (b) Renal angiomyolipoma, (c) Spontaneous rupture, (d) Ventricular tachycardia

## Abstract

**a) Introduction and Importance:**

Angiomyolipomas of kidney are benign lesions that are generally an incidental finding on imaging. Rupture of angiomyolipoma is rare and fatal complication that requires early intervention.

**b) Case Presentation:**

A 38 year old male patient presented with symptoms of right flank pain for 2 days. On clinical examination patient looked anxious, pale with right flank tenderness, guarding and signs of shock.

**c) Clinical findings and investigations:**

CT scan showed renal angiomyolipoma with aneurysm formation and bleed from the lesion. Blood profile revealed low hemoglobin.

**d) Interventions and Outcome:**

Right nephrectomy performed along 6 cycles of cardiopulmonary reususcitaion done with stable post operatively vitals.

**e) Conclusion:**

Early diagnosis of complication of angiomyolipoma requires thorough clinical examination and judicious use of imaging. Immediate embolization or surgery must be performed for better outcome and survival rate.

## Highlights

• Angiomyolipoma is composed of fat, smooth muscle and blood vessels and presents among 0.3% of the population.
• Renal angiomyolipoma are prone to aneurysm formation and bleed, however a nontraumatic spontaneous renal hemorrhage from a sporadic renal angiomyolipoma is a rare condition.
• Mostly angiomyolipoma are asymptomatic and are diagnosed incidentally. CT scan is the diagnostic modality of choice.
• Patients with life-threatening hemorrhage require immediate intervention that includes embolization or surgery for better outcome and survival rate.

## 1 Introduction

Angiomyolipomas (AMLs), which belongs to a family of tumors collectively called neoplasms with perivascular epithelioid differentiation (PEComa), is composed of fat, smooth muscle and blood vessels [1,2]. AML generally presents among 0.3% of the population [[3], [4], [5]] and commonly affects female in their 5th decade of their life [1,6]. AMLs are mostly diagnosed incidentally by imaging [1,6]. However renal AML can clinically present with flank pain (40–50%), palpable mass, hematuria and shock [6,7]. AML presented with shock requires early embolization and surgical intervention for better survival rate [7,8]. Few cases of spontaneous rupture of AML have been reported [9,10].

Herein we report a rare and fatal case of spontaneous nontraumatic rupture of AML that was successfully managed with surgical removal of the tumor along with nephrectomy. This case report has been reported in line with the SCARE 2020 criteria [11].

## 2 Method

We reported this case following the updated consensus-based Surgical Case Report (SCARE) Guidelines [11].

## 3 Case Presentation

A 38 year old male army personnel with no known co-morbidities was referred to our hospital with complaints of right flank pain for last 2 days. The pain was pricking in nature, localized to right flank, associated with decreased frequency of micturation and no known aggravating factors. There was no history of any trauma, fever, vomiting. On clinical examination the patient looked anxious, pale with tenderness over the right flank and guarding but no rebound tenderness. His past history, family history and allegric history were non remarkable. He is a non smoker, doesnot consume alcohol and has a normal bowel habit.

On examining his vital parameters, he had low blood pressure (90/60 mmHg, measured on lying) and low SpO2: 89% with elevated pulse rate: 130 per min. The laboratory analyses were sent and it showed decreased level of hemoglobin (Hb): 8.6 gm/dl along with elevated total leucocyte count (TLC): 13500 cells/cumm, neutrophils: 80%, urea: 75mg/dl and creatinine: 2.66mg/dl. His prothrombin time was 12.5 seconds, INR: 0.9, Random Blood Sugar (RBS): 133 and serology was non-reactive for HIV, Hepatitis B and Hepatitis C.

**Fig. 1:**
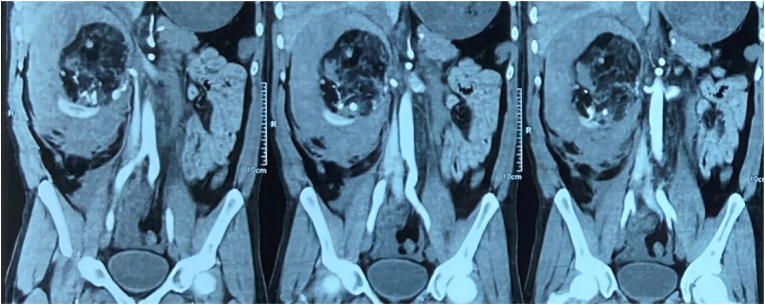
Contrast Enhanced Computerized Tomography (CECT) of abdomen and pelvis (Coronal section) showing right angiomyolipoma.

**Fig. 2:**
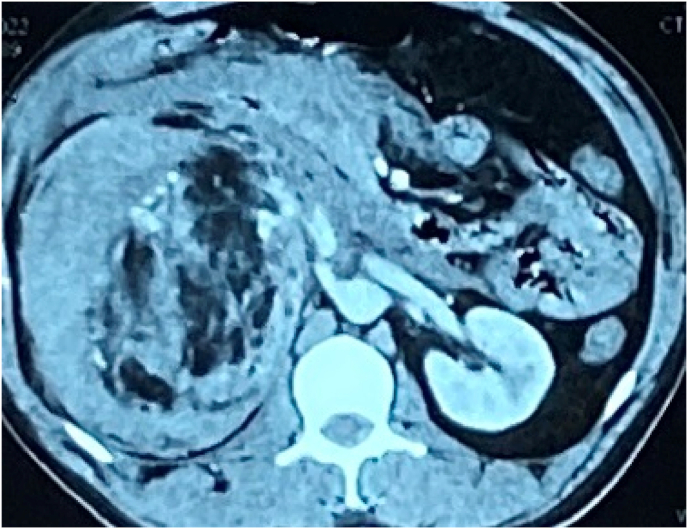
Contrast Enhanced Computerized Tomography (CECT) of abdomen and pelvis (axial section) showing right angiomyolipoma.

Ultrasonography (USG) of abdomen was ordered which showed heterogenous lesion with cystic space. Also, CT scan reports revealed angiomyolipoma (measuring 13.5 × 10.4 × 13cm) arising from upper pole of right kidney. In arterial phase of scan, multiple aneurysms were noted within the lesion, largest measuring 12 × 8mm. Features indicating acute bleed from renal angiomyolipoma were also visualized (Fig. 1, Fig. 2).

**Fig. 3:**
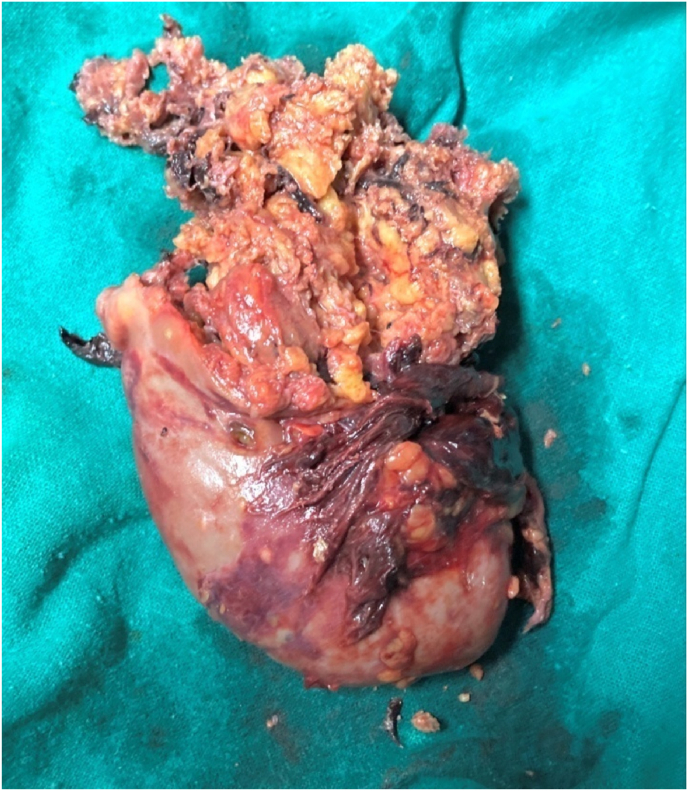
Gross specimen of right kidney along with tumor postoperatively.

The patient was in hypovolemic shock and thus fluid resuscitation with 1 L normal saline along with transfusion of one pint of packed red blood cells (PRBC) was done in the emergency room. The patient was then rushed to the operation theatre and underwent exploratory laparotomy with removal of tumor along with nephrectomy of right kidney (Fig. 3). Intraoperatively, lipomatous tumor in right kidney, 15cm × 10 cm retroperitoneal hematoma, 300ml of intraperitoneal hematoma and 1 L of clots were noticed. Two additional pints of packed red blood cells (PRBC) and two pints of fresh frozen plasma (FFP) were transfused intraoperatively.

At the end of the surgery, patient went to ventricular tachycardia. Immediate cardiopulmonary resuscitation (CPR) was initiated along with DC shock aided with 1mg IV adrenaline, 64mg IV xylocard (lignocaine) and 300mg IV amiodarone with 150mg maintenance. A total of 6 cycles of CPR was given and Return of spontaneous circulation (ROSC) was observed. Hyperkalemia was suspected to be the cause of arrhythmia as a complication of repeated blood transfusion which was confirmed by arterial blood gas (ABG) analysis report that showed low pH: 7.2 and increased level of potassium: 6.8mmol/l. So,Post return of spontaneous circulation (ROSC) was accompanied by calcium gluconate 10ml of 10% and 10 units of insulin with 50ml of 50% dextrose. His random blood sugar was noted to be 265mg/dl.

After completion of surgery, he was shifted to surgical ICU with continued mechanical ventilation and inotropic support. He was later extubated on 1st post operative day. Later on that same day, his postoperative haemoglobin was ordered which showed that he was anemic (Hb: 7.8 gm/dl) and thus was followed by transfusion of two pint of packed red blood cells (PRBC). On 2nd post operative day, inotropic supports were stopped. The histopathological examination was sent which established the diagnosis of right angiomyolipoma (Classical variant). On day 12, the patient was asymptomatic and hemodynamically stable with normal renal function tests, so he was discharged from the hospital.

Follow up was carried out 1 month post hospitalization in outpatient department and that the patient is currently asymptomatic.

## 4 Discussion

Most renal angiomyolipoma are presented asymptomatically and were found to have evidence of renal mass on cross-sectional imaging [3]. But patients may present with symptoms like flank pain, hematuria and palpable mass including less frequent symptoms like nausea, vomiting, fever, anaemia and blood pressure alteration. Sporadic AMLs often present among woman unilaterally with slow growth rate and express preference for 50–60 years of age [12]. 20% of the AML coexist in patients with tuberous sclerosis where the lesion grows more faster and present bilaterally in an younger age group [12]. Patient with tuberous sclerosis manifest cognitive dysfunction, seizure and skin lesions [13,14]and require survillence starting from younger age and continuing even in latter ages of life as it possess significant mortality and morbidity [4]. However our patient was not a case of tuberous sclerosis.

Renal AMLs are prone to aneurysm formation and hemorrhage due to hypervascularity with tortuous blood supplies and the lack of elastic membrane [15,16]. Tumor size greater than 4 cm is considered as a predictor of rupture however if aneurysm size greater than 5mm is considered, the specificity increases [12,17].

Initial evaluation includes clinical signs/symptoms, blood investigations and imaging. On ultrasound, classic AML is hyperechoic indistinguishable from Renal cell carcinoma (RCC) [18]. The imaging of choice for AML is considered to be Computed Tomography scans. In case of classic AML, detection of fat is a identifying feature [18]. When negative 20 HU or less is recorded within a renal lesion on non – enhanced CT scan, it is considered diagnostic hall mark [9,19]. In case of fat poor and fat invisible AML the CT scan shows homogenously hyperattenuating lesion [19]. MRI can also be useful in detecting fat however not used as a diagnostic choice, as AML can be hard to differentiate form any other renal tumor with bleed [9]. Also in MRI, fat cells are hard to spot from other cells that contain intracytoplasmic fats [18]. When AML cannot be distinguished from RCC in any radiological modalities, mostly when the fat content is poor [18], percutaneous biopsy is recommended [19].

AMLs with minimal risk of bleed are generally managed through active surveillance [7,15]. Embolization is recommended in AML with high risk of bleed as it can help preserve renal function and avoid surgery and anesthesia. Embolization reduces the risk of hemorrhage by blocking blood supply to the AML. Patients may experience post embolization syndrome due to an inflammatory response to necrotic tissue that includes flank pain, fever, leukocytosis and nausea [8,15]. Also, there is notable risk of recurrent bleed and relapse [4,6]. There are documentation of re-embolization and secondary surgery in 30–50% of patient treated with embolization [3,12,14]. Complication in 10% of case including a case of death was noted in emobilzation [8].

Partial or total nephrectomy can only be justified if one suspect malignancy or in patient with bleed to control the hemorrhage, if embolization is unavailable or unsuccessful [8]. There has not been any documentation of recurrence experienced in patient that underwent surgery [3]. In addition, nephron sparing surgery and Radiofrequency ablation (RFA) are also considered in AML.

Our patient also experienced ventricular tachycardia which was confirmed through characteristic electricardiographic changes. Ventricular tachycardia consists of several contributing factors, one of which includes hyperkalemia. Management of electrolyte imbalance can reverse the arrhythmia [20].

## 5 Conclusion

The main complication of AML is tumor rupture resulting in retroperitoneal hemorrhage which can be life threatening. Clinician should keep a high index of suspicion and thorough clinical examination. Prompt diagnosis can be made through computed tomography scan. Patients with life-threatening hemorrhage require timely intervention that includes embolization or surgery for better outcome and for lowering mortality rate.

## Ethical approval

Not required.

## Sources of funding

No any funding for our manuscript.

## Author contribution

Narayan Thapa, Suman Maharjan, Anil Hona, Sabin Karki and Jayaram Pandey were involved in writing, editing and review of the manuscript. Narayan Thapa, Anil Hona and Suman Maharjan were involved in the management of the patient. All authors read and approved the final manuscript.

## Registration of research studies

Name of the registry:

Unique Identifying number or registration ID:

Hyperlink to your specific registration (must be publicly accessible and will be checked):

## Guarantor

Suman Maharjan.

## Consent

Written informed consent was obtained from the patient for publication of this case report and accompanying images. A copy is available for review by the Editor in chief of this journal on request.

## Funding

No funding was required for the publication of this case report.

## Written consent from the patient

Written informed consent was obtained from the patient for publication of this case report and accompanying images. A copy is available for review by the Editor in chief of this journal on request.

## Data availability statement

Not applicable.

## Provenance and peer review

Not commissioned, externally peer reviewed.

## Declaration of competing interest

No any potential conflict of interest and no any financial and personal relationship with others that could influence the manuscript.

## References

[1] Ng K.F., Chen T.C. (2001). Infiltrating renal angiomyolipoma into ascending colon associated with hepatic involvement in a patient with tuberous sclerosis. J. Urol..

[2] Idilman I.S., Vesnic S., Cil B., Peynircioglu B. (2014). Giant renal artery pseudoaneurysm caused by rupture of renal angiomyolipoma following pregnancy: endovascular treatment and review of the literature. Saudi J Kidney Dis Transpl.

[3] Mues A.C., Palacios J.M., Haramis G., Casazza C., Badani K., Gupta M. (2010). Contemporary experience in the management of angiomyolipoma. J. Endourol..

[4] Esmat H.A., Naseri M.W. (2021). Giant renal pseudoaneurysm complicating angiomyolipoma in a patient with tuberous sclerosis complex: an unusual case report and review of the literature. Ann Med Surg.

[5] Lemaitre L., Claudon M., Dubrulle F., Mazeman E. (1997). Imaging of angiomyolipomas. Semin. Ultrasound CT MRI.

[6] Wang C., Li X., Peng L., Gou X., Fan J. (2018). An update on recent developments in rupture of renal angiomyolipoma. Med (United States).

[7] Oesterling J.E., Fishman E.K., Goldman S.M., Marshall F.F. (1986). The management of renal angiomyolipoma. J. Urol..

[8] Nelson C.P., Sanda M.G. (2002). Contemporary diagnosis and management of renal angiomyolipoma. J. Urol..

[9] Chronopoulos P.N., Kaisidis G.N., Vaiopoulos C.K., Perits D.M., Varvarousis M.N., Malioris A.V. (2016). Spontaneous rupture of a giant renal angiomyolipoma - wunderlich's syndrome: report of a case. Int J Surg Case Rep.

[10] Altuwayr R.M., Almutairi F.S., Alkhaibari S.H., Alharbi A.M., Alramih A.A., Alamri R.A. (2021). Spontaneous rupture of large angiomyolipoma of the kidney: a rare case. Cureus.

[11] Agha R.A., Franchi T., Sohrabi C., Mathew G., Kerwan A., Thoma A. (2020). The SCARE 2020 guideline: updating consensus surgical CAse REport (SCARE) Guidelines. Int. J. Surg..

[12] Fernández-Pello S., Hora M., Kuusk T., Tahbaz R., Dabestani S., Abu-Ghanem Y. (2020). Management of sporadic renal angiomyolipomas: a systematic review of available evidence to guide recommendations from the European association of urology renal cell carcinoma Guidelines panel. Eur Urol Oncol.

[13] Caliò A., Brunelli M., Segala D., Zamboni G., Bonetti F., Pea M. (2021). Angiomyolipoma of the kidney: from simple hamartoma to complex tumour. Pathology.

[14] Harabayashi T., Shinohara N., Katano H., Nonomura K., Shimizu T., Koyanagi T. (2004). Management of renal angiomyolipomas associated with tuberous sclerosis complex. J. Urol..

[15] Halpenny D., Snow A., McNeill G., Torreggiani W.C. (2010). The radiological diagnosis and treatment of renal angiomyolipoma-current status. Clin. Radiol..

[16] Salık A.E. (2019). Transarterial embolization of symptomatic renal angiomyolipomas. Med J Bakirkoy.

[17] Yamakado K, Tanaka N, Nakagawa T, Kobayashi S, Yanagawa M. Angimiolipomi Rottura n.d.

[18] Jinzaki M., Silverman S.G., Akita H., Nagashima Y., Mikami S., Oya M. (2014). Renal angiomyolipoma: a radiological classification and update on recent developments in diagnosis and management. Abdom. Imag..

[19] Park B.K. (2017). Renal angiomyolipoma: radiologic classification and imaging features according to the amount of fat. Am. J. Roentgenol..

[20] Seto Atsushi, Murakami Masako, Fukuyama Hiroshi, Niijima Kuniyuki, Aoyama Kazuyoshi, Ichiro Takenaka T.K. (2000). Ventricular tachycardia caused by hyperkalemia after administration of hypertonic mannitol. Anesthesiology.

